# Prevalence and Risk Factors for COPD at High Altitude: A Large Cross-Sectional Survey of Subjects Living Between 2,100–4,700 m Above Sea Level

**DOI:** 10.3389/fmed.2020.581763

**Published:** 2020-12-03

**Authors:** Yanfei Guo, Zhenzhen Xing, Guangliang Shan, Jean-Paul Janssens, Tieying Sun, Di Chai, Weiming Liu, Yuxia Wang, Yali Ma, Yaqi Tong, Yilin Huang, Yang Cao, Chen Wang

**Affiliations:** ^1^National Clinical Research Center for Respiratory Diseases, Beijing, China; ^2^Department of Respiratory and Critical Care Medicine, Beijing Hospital, Beijing, China; ^3^National Center of Gerontology, Beijing, China; ^4^School of Basic Medicine, Institute of Basic Medical Sciences, Peking Union Medical College, Chinese Academy of Medical Sciences, Beijing, China; ^5^Division of Pulmonary Diseases, Department of Medicine, Geneva University Hospitals, Geneva, Switzerland; ^6^Department of Intensive Care Medicine, Beijing Boai Hospital, Beijing, China; ^7^Rehabilitation Research Center, Beijing, China; ^8^Department of Pulmonary and Critical Care Medicine, Center of Respiratory Medicine, China-Japan Friendship Hospital, Beijing, China; ^9^Chinese Academy of Medical Sciences and Peking Union Medical College, Beijing, China; ^10^Department of Respiratory Medicine, Capital Medical University, Beijing, China; ^11^WHO Collaboration Center for Tobacco Cessation and Respiratory Diseases Prevention, Beijing, China

**Keywords:** risk factors, prevalence, tuberculosis, high altitude, household air pollution, Chronic Obstructive Pulmonary Disease (COPD)

## Abstract

**Aim of Study:** Four hundred million people live at high altitude worldwide. Prevalence and risk factors for COPD in these populations are poorly documented. We examined the prevalence and risk factors for COPD in residents living at an altitude of 2,100–4,700 m.

**Methods:** We performed a cross-sectional survey in Xinjiang and Tibet autonomous region. A multistage stratified sampling procedure was used to select a representative population aged 15 years or older from eight high altitude regions. All participants underwent pre- and post-bronchodilator measurement of forced expiratory volumes. COPD was diagnosed according to 2019 Global Initiative for Chronic Obstructive Lung Disease (GOLD) criteria.

**Results:** Between June, 2015 and August 2016, 4,967 subjects were included. Median age was 38.0 years (range: 15–91 years; inter-quartile range: 28–49 years); 51.4% participants were female. Overall prevalence of spirometry-defined COPD was 8.2% (95% CI 7.4–8.9%): 9.3% in male (95% CI 8.2–10.4%), and 7.1% in female (95% CI 6.1–8.2%). By multivariable logistic regression analysis, COPD was significantly associated with being aged ≥40 years (odds ratio: 2.25 [95% CI 1.72–2.95], *P* < 0.0001), exposure to household air pollution (OR: 1.34 [95% CI 1.01–1.79], *P* = 0.043), and a history of tuberculosis (OR: 1.79 [95% CI 1.23–2.61], *P* = 0.030), while living at a higher altitude (OR: 0.45 [95% CI 0.33–0.61], *P* < 0.0001) and having a higher educational level (OR: 0.64 [95% CI 0.43–0.95], *P* = 0.025) were associated with a lower prevalence of COPD.

**Conclusions:** Our results show that the spirometry-defined COPD is a considerable health problem for residents living at high altitudes and COPD prevalence was inversely correlated with altitude. Preventing exposure to household air pollution and reducing the incidence of tuberculosis should be public health priorities for high altitude residents.

## Introduction

Chronic obstructive pulmonary disease (COPD) is one of the leading causes of death from non-communicable diseases in the world, accounting for 5.4% of all deaths globally in 2016 (1–3). In China, COPD has been one of the top four causes of death over the past 30 years (4). The Global Burden of Disease study estimated that there were 299 million adults with prevalent COPD worldwide in 2017 (5), and 99.9 million individuals aged ≥20 years with COPD in China in 2015 (6), causing a substantial clinical, economic, and societal burden. Globally, around 400 million people live at high altitude (defined as >1,500 m above sea-level) (7). However, to date, few studies have focused on the prevalence of COPD in high altitude regions, especially for areas with altitudes above 4,000 m.

Living at high altitude results in exposure to colder temperatures, a lower humidity, and hypobaric hypoxic conditions. These conditions could induce physiological adaptations such as changes in lung volumes or diffusing capacity in residents at high altitude (8). At 4,000 m, ambient arterial partial pressure of oxygen (PaO_2_) is ~63% that of PaO_2_ measured at sea level (9, 10). These variables may impact on respiratory health in general and prevalence of COPD in particular. Approximately 90% of deaths from COPD occur in low- and middle-income countries (11). Thus the high altitude setting combines social-economic factors and environmental conditions which may affect respiratory health.

In the present study, we aimed to determine the prevalence of COPD and to explore potential risk factors for COPD in residents living at high altitude.

## Methods

### Study Design and Participants

Between June, 2015 and August 2016, a multistage stratified sampling procedure was used to select a representative sample of subjects living at an altitude of 2,100–4,700 m. Firstly, eight districts or counties were selected in both urban (2 districts) and rural (6 counties) areas using the probability proportional to size method. Lhasa Chengguan District (altitude 3,650 m above sea level) and Shigatse City (altitude 3,900 m) were selected as urban sites. Six counties were also selected: Linzhi County (altitude 3,000 m), Anduo County (4,700 m), Xietongmen County (4,100 m), Duilongdeqing County (4,500 m), Aheqi County (2,100 m) and Tashkurgan County (3,200 m). Subsequently, using a simple random sampling method, two streets or townships were selected from each district or county, and three communities or village communities were selected from each street or township. Finally, using the same method, we chose participants from each of the sex/age strata from communities or villages. The proportion of samples from each gender and age group was based on the 2010 census of Chinese population. We selected only one participant from each household. We included permanent residents (i.e., living in their current residence for more than 1 year) aged ≥15 years for this analysis.

Exclusion criteria were: treatment for tuberculosis during study period; myocardial infarction or cerebrovascular accident during the previous 3 months; pregnancy; heart rate >120 beats/min or blood pressure >180/120 mmHg, and any condition that would impede the use of spirometry (such as recent thoracic, abdominal, or eye surgery, or retinal detachment).

The study protocol was approved by the Institutional Review Board and ethics committee of Beijing Hospital (2013BJYYEC-042C-01). Each participant received detailed information about the study and study methods, and provided written informed consent before data collection.

### Procedures

A standardized questionnaire covering demographic data, living conditions, respiratory symptoms, history of respiratory diseases and comorbidities, and potential risk factors for COPD was administered by experienced interviewers. Forced expiratory volumes were measured in all qualified study participants (spirometry) with a MasterScreen™ Pneumo PC spirometer (CareFusion, Yorba Linda, CA) according to ATS/ERS recommendations (12). The spirometer was calibrated daily using a 3 L syringe to ensure measured volumes within 3% of syringe volume, before data collection; ambient temperature, humidity, and altitude were also recorded daily. Each participant underwent the same procedure twice, before and after receiving a bronchodilator (400 ug of salbutamol through a 500 ml spacer). The forced expiratory maneuvers were performed 3–8 times until the forced vital capacity (FVC) and forced expiratory volume in 1 s (FEV_1_) were reproducible within 150 ml. Acceptability of FVC and FEV_1_ was scored using the grading system (A-F) of Enright et al. (13). Performing 3 acceptable maneuvers with an FVC variability of 100 ml or less was rated “A”; a variability of 100–150 ml was rated “B”; variability between 150 and 200 ml was scored “C.” A, B, or C grades were considered acceptable for analysis. Data were uploaded daily to a database, examined for incoherent data by the study supervisors and by the principal investigator. Quality control, based on the American Thoracic Society/European Respiratory Society criteria, was performed by a field supervisor at the filing center (12).

COPD was defined as a post-bronchodilator FEV_1_/FVC ratio of <0.70, based on Global Initiative for Chronic Obstructive Lung Disease (GOLD) guidelines (14). The ratios of observed/predicted FEV_1_ based on a nationwide study of reference values for spirometry in the Chinese population were used to stage the degree of obstruction: GOLD grade I ≥80% predicted, GOLD grade II 50–79% predicted, GOLD grade III 30–49% predicted, and GOLD grade IV <30% predicted (15). The lower limit of normal (LLN) of reference values was also used to define COPD in a sensitivity analysis based on GLI lung function equations for a South East Asian population (16). Awareness of COPD was defined as self-reported physician-diagnosed COPD among patients with COPD.

Exposure to household air pollution was defined as the use of wood, animal waste, or coal for cooking or heating during the previous 6 months or longer. Peripheral oxygen saturation was measured with a pulse-oximetry (PHILIPS DB12) before performing spirometry. Smoking status was categorized as: current smoker, former smoker, or never smoker. We defined current smokers as active smokers who had smoked more than 100 cigarettes in their life time; former smokers had smoked more than 100 cigarettes in their lifetime but had stopped smoking at least 12 months before the interview (17). The mean altitudes of the six study areas were obtained from the local administrative departments.

### Statistical Analysis

Our study was designed to provide reliable estimates of the prevalence of COPD for both male and female from five age groups (15–29, 30–39, 40–49, 50–59, and ≥60 years) in rural and urban settings. The sample sizes were calculated separately for populations aged 15–39 and ≥40 years due to large differences in prevalence. Based on available data from previous studies, we assumed a COPD prevalence of 3.0% in the 15–39-year age group and 9.9% in the ≥40-year age group (18, 19). In addition, we used a design effect of 1.5 (the ratio of the variance of a statistic from a complex sample to the variance of the same statistic from a simple random sample of the same size) to account for the multistage cluster sampling design. The final sample sizes were 1,735 in the 15–39 years age group and 3,312 in the ≥40 years age group. Demographic data are expressed as mean ± SD for continuous variables, then compared by analysis of variance (ANOVA) or *t*-tests as appropriate. Data for categorical variables were presented as counts and proportions, and were compared by χ^2^-tests. Multivariable logistic regression analyses were conducted to explore risk factors (sex, age, smoking status, region, altitude, educational level, household air pollution, history of tuberculosis, and exposure to dust or chemicals in the workplace) for COPD in all study participants. Differences with two-sided *P* < 0.05 were considered statistically significant. All statistical analyses were performed using SAS version 9.4 (SAS Institute Inc., Cary, North Carolina).

## Results

**Figure 1:**
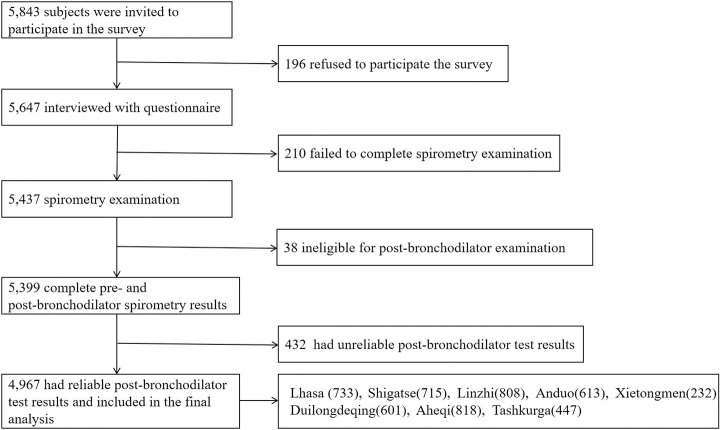
Flow chart of study.

To meet the designed sample size, a total of 5,843 permanent residents were randomly selected and 5,647 participants were recruited. After excluding individuals with unreliable post-bronchodilator tests, 4,967 participants aged ≥15 years (2,415 male and 2,552 female) were included in the final analysis and completed the survey (Figure 1).

Table 1 summarizes the characteristics of individuals sorted by three different altitudes of residence: 2,100–3,000, 3,000–4,000, and >4,000 m. Educational level of men was higher than that of women for all subjects. Educational level was lower in subjects living at higher altitude. Proportion of current and former smokers was lower in men living at very high altitude (>4,000 m). Among male smokers, the proportion of heavy smokers (≥20 pack-years) living at 2,100–3,000 m was more than twice that of those living above 4,000 m (58.5 vs. 20.1%). Exposure to household air pollution (3,370 subjects; 67.8%), was more important in the female population (73.0%) than in males (62.4%). Subjects living in rural regions (2,100–3,000 and >4,000 m) were more exposed to household air pollution. Conversely, participants living in cities (3,000–4,000 m) were more exposure to dust or chemicals in the workplace and had a higher prevalence of history of tuberculosis. As expected, oxyhemoglobin saturation (SpO_2_) of participants decreased with altitude.

**Table 1:** Demographic characteristics of high-altitude residents by altitude and gender: anthropometric data, education, smoking history, risk factors and results of pulmonary function tests.

**Variables**	**Total (*n* = 4,967)**				**Men (*n* = 2,415)**				**Women (*n* = 2,552)**			
	**2,100–3,000 m (*n* = 1,626)**	**3,000–4,000 m (*n* = 1,895)**	**>4,000 m (*n* = 1,446)**	***P-value***	**2,100–3,000 m (*n* = 807)**	**3,000–4,000 m (*n* = 956)**	**>4,000 m (*n* = 652)**	***P-value***	**2,100–3,000 m (*n* = 819)**	**3,000–4,000 m (*n* = 939)**	**>4,000 m (*n* = 794)**	***P-value***
**Age, years**												
15–39	898 (55.3%)	1,038 (54.7%)	723 (50.0%)	<0.0001	462 (57.2%)	605 (63.3%)	335 (51.4%)	<0.0001	436 (53.2%)	433 (46.1%)	388 (48.9%)	0.002
40–49	348 (21.4%)	382 (20.2%)	382 (26.4%)		162 (20.1%)	158 (16.5%)	175 (26.8%)		186 (22.7%)	224 (23.9%)	207 (26.1%)	
50–59	233 (14.3%)	271 (14.3%)	247 (17.1%)		104 (12.9%)	104 (10.9%)	105 (16.1%)		129 (15.8%)	167 (17.8%)	142 (17.9%)	
≥60	147 (9.0%)	204 (10.8%)	94 (6.5%)		79 (9.8%)	89 (9.3%)	37 (5.7%)		68 (8.3%)	115 (12.2%)	57 (7.2%)	
Body-mass index, kg/m^2^	23.4 (3.7)	23.7 (3.9)	24.0 (4.2)	<0.0001	23.3 (3.6)	23.5 (3.5)	24.0 (4.0)	0.001	23.4 (3.8)	23.9 (4.4)	24.0 (4.3)	0.022
**Education level**												
Primary school and lower	812 (49.9%)	863 (45.5%)	993 (68.7%)	<0.0001	332 (41.1%)	295 (30.9%)	421 (64.6%)	<0.0001	480 (58.6%)	568 (60.5%)	572 (72.0%)	<0.0001
Middle and high school	608 (37.4%)	473 (25.0%)	245 (16.9%)		341 (42.3%)	280 (29.3%)	126 (19.3%)		267 (32.6%)	193 (20.6%)	119 (15.0%)	
College and higher	206 (12.7%)	559 (29.5%)	208 (14.4%)		134 (16.6%)	381 (39.9%)	105 (16.1%)		72 (8.8%)	178 (19.0%)	103 (13.0%)	
**Smoking status**												
Never smoker	1,071 (65.9%)	1,371 (72.3%)	1,162 (80.4%)	<0.0001	317 (39.3%)	445 (46.5%)	471 (72.2%)	<0.0001	754 (92.1%)	926 (98.6%)	691 (87.0%)	<0.0001
Former smoker	112 (6.9%)	81 (4.3%)	107 (7.4%)		101 (12.5%)	76 (8.0%)	60 (9.2%)		11 (1.3%)	5 (0.5%)	47 (5.9%)	
Current smoker	443 (27.2%)	443 (23.4%)	177 (12.2%)		389 (48.2%)	435 (45.5%)	121 (18.6%)		54 (6.6%)	8 (0.9%)	56 (7.1%)	
Cigarette smoking, pack-year^*^												
0	1,071 (65.9%)	1,371 (72.5%)	1,162 (80.6%)	<0.0001	317 (39.4%)	445 (46.7%)	471 (72.6%)	<0.0001	754 (92.1%)	926 (98.7%)	691 (87.0%)	<0.0001
1–19	19 (1.2%)	50 (2.6%)	68 (4.7%)		17 (2.1%)	49 (5.1%)	47 (7.3%)		2 (0.2%)	1 (0.1%)	21 (2.6%)	
≥20	534 (32.9%)	470 (24.9%)	212 (14.7%)		471 (58.5%)	459 (48.2%)	130 (20.1%)		63 (7.7%)	11 (1.2%)	82 (10.3%)	
**Risk factors for COPD**												
Household air pollution^*^	1,356 (83.4%)	1,070 (56.5%)	944 (66.0%)	<0.0001	626 (77.6%)	472 (49.4%)	408 (63.6%)	<0.0001	730 (89.1%)	598 (63.7%)	536 (67.9%)	<0.0001
History of tuberculosis	82 (5.0%)	122 (6.4%)	50 (3.5%)	0.001	40 (5.0%)	52 (5.4%)	27 (4.1%)	0.497	42 (5.1%)	70 (7.5%)	23 (2.9%)	<0.0001
Exposure to dust or chemicals in the workplace^*^	14 (0.9%)	109 (5.9%)	15 (1.0%)	<0.0001	8 (1.0%)	54 (5.8%)	10 (1.5%)	<0.0001	6 (0.7%)	55 (6.0%)	5 (0.6%)	<0.0001
**Spirometry,%**												
Pre-BD FEV_1_/FVC	79.34 (10.07)	84.38 (9.84)	84.56 (10.26)	<0.0001	79.10 (10.40)	84.61 (9.94)	84.36 (9.96)	<0.0001	79.58 (9.74)	84.16 (9.73)	84.72 (10.50)	<0.0001
Post-BD FEV_1_/FVC	81.7 (10.0)	85.75 (9.30)	85.75 (9.47)	<0.0001	81.20 (10.41)	86.05 (9.23)	85.19 (10.14)	<0.0001	82.24 (9.57)	85.45 (9.37)	86.21 (8.86)	<0.0001
Pre-BD FVC %predicted	79.33 (10.08)	84.37 (9.84)	84.55 (10.26)	<0.0001	79.08 (10.40)	84.60 (9.94)	84.36 (9.96)	<0.0001	79.57 (9.75)	84.14 (9.73)	84.71 (10.51)	<0.0001
Post-BD FVC %predicted	82.07 (11.45)	85.88 (13.05)	86.25 (22.25)	<0.0001	81.75 (11.60)	86.43 (13.36)	85.00 (15.15)	<0.0001	82.38 (11.29)	85.33 (12.71)	87.28 (26.68)	<0.0001
Pre-BD FEV_1_ %predicted	104.11 (13.47)	102.22 (15.02)	102.36 (24.07)	0.003	104.06 (13.68)	102.70 (16.51)	100.92 (14.76)	<0.0001	104.15 (13.26)	101.74 (13.32)	103.55 (29.56)	0.029
Post-BD FEV_1_ %predicted	103.70 (11.74)	102.26 (12.30)	102.42 (15.76)	0.003	103.37 (11.74)	102.41 (12.67)	101.65 (13.80)	0.034	104.03 (11.75)	102.12 (11.93)	103.05 (17.19)	0.014
Oxyhemoglobin saturation, %	93.5 (3.3)	90.6 (5.1)	86.3 (4.5)	<0.0001	93.1 (3.1)	90.6 (4.9)	86.2 (4.6)	<0.0001	94.0 (3.3)	90.6 (5.3)	86.4 (4.4)	<0.0001

*Data are expressed as number (%) or mean (standard deviation, SD). COPD, chronic obstructive pulmonary disease; FVC, forced vital capacity; FEV1, forced expiratory volume in the first second; Pre-BD, pre-bronchodilator; Post-BD, post-bronchodilator*.

* *assessed in Cigarette smoking with data missing for 10 participants, household air pollution with data missing for 15 participants and exposure to dust or chemicals in the workplace with data missing for 38 participants*.

**Figure 2:**
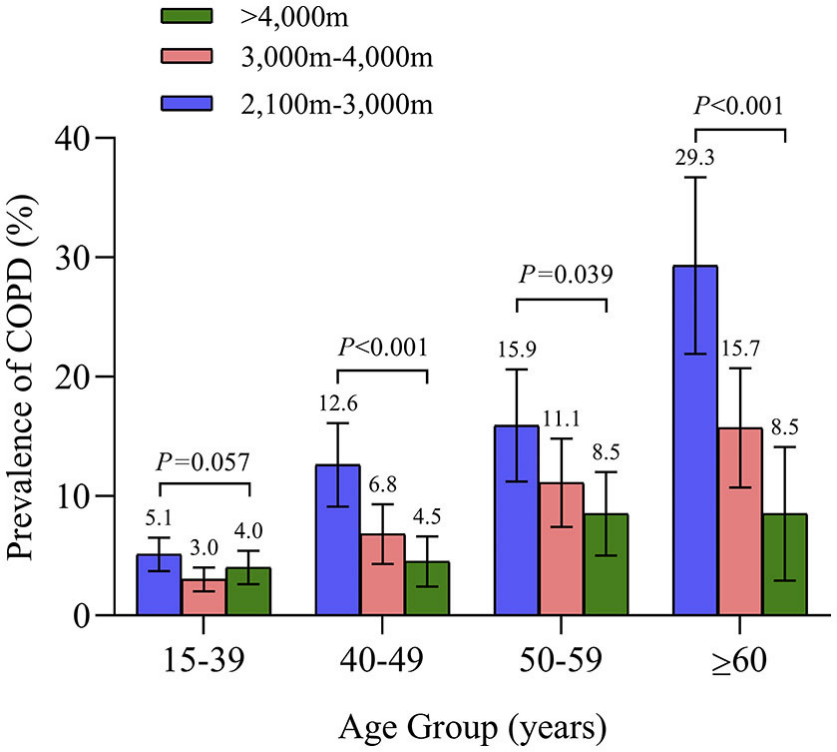
Prevalence of Chronic Obstructive Pulmonary Disease in each age group according to altitude of residence.

A total of 364 participants in the survey had an FEV_1_/FVC ratio of <70% and were diagnosed with COPD (Table 2). The overall standardized prevalence of spirometry-defined COPD (GOLD diagnostic criteria) was 8.2% (95% CI 7.4–8.9%); difference between genders was not significant. Prevalence increased with age (Figure 2); it reached 12.3% (10.9–13.7%; *P* < 0.0001 for age difference) among those aged ≥40 years. Conversely, as shown in Figure 2, prevalence of COPD decreased significantly with increasing altitude. Supplementary Table 1 shows GOLD stage of the COPD population according to different altitudes. The prevalence of respiratory symptoms (i.e., cough, sputum, wheezing, and dyspnea) in patients with COPD is shown in the Table 3. Overall, more than a half of individuals with COPD had at least one respiratory symptom (57.9%), but 42.1% had no symptoms suggestive of COPD. The frequency of respiratory symptoms increased with GOLD severity stage. Among COPD with a completed CAT score (360), 87.2% had a score of 10 or higher. Only 1.1% of patients diagnosed with COPD were previously aware of their diagnosis. Of all patients with COPD, none had ever been tested by spirometry before the survey.

**Table 2:** Age-specific and age-standardized prevalence (%) of chronic obstructive pulmonary disease in the residents aged 15 years older living at high altitude.

**Variables**	**Overall**		**Men**		**Women**	
	**Cases/total (*n*/*N*)**	**Prevalence of COPD (95%CI)**	**Cases/total (*n*/*N*)**	**Prevalence of COPD (95%CI)**	**Cases/total (*n*/*N*)**	**Prevalence of COPD (95%CI)**
Prevalence	364/4,967	8.2% (7.4–8.9)	187/2,415	9.3% (8.2–10.4)	177/2,552	7.1% (6.1–8.2)
**Age, year**						
15–39	106/2,659	3.7% (2.9–4.4)	59/1,402	3.6% (2.5–4.6)	47/1,257	3.7% (2.6–4.7)
40–49	87/1,112	7.2% (5.6–8.9)	42/495	8.7% (6.1–11.2)	45/617	6.2% (4.1–8.3)
50–59	88/751	11.1% (8.7–13.6)	34/313	12.2% (8.5–15.8)	54/438	10.3% (7.0–13.5)
≥60	83/445	33.3% (29.3–37.3)	52/205	46.2% (39.3–53.1)	31/240	22.6% (18.0–27.1)
*P for trend*		<0.0001		<0.0001		<0.0001
**Altitude, m**						
2,100–3,000	170/1,626	12.1% (10.5–13.7)	95/807	13.9% (11.5–16.3)	75/819	10.1% (8.0–12.1)
3,000–4,000	119/1,895	6.9% (5.8–8.0)	52/956	6.7% (5.2–8.1)	67/939	7.0% (5.3–8.7)
>4,000	75/1,446	5.4% (4.2–6.6)	40/652	7.1% (5.2–8.9)	35/794	4.2% (2.8–5.7)
*P for trend*		<0.0001		<0.0001		<0.0001
**Education level**						
Primary school and lower	269/2,668	8.8% (7.5–10.0)	125/1,048	10.3% (8.2–12.4)	144/1,620	7.6% (6.2–9.1)
Middle and high school	55/1,326	6.3% (5.3–7.4)	38/747	8.4% (6.7–10.0)	17/579	2.6% (1.2–4.0)
College and higher	40/973	6.0% (4.7–7.2)	24/620	5.5% (3.9–7.0)	16/353	6.8% (4.6–9.0)
*P for trend*		<0.0001		<0.0001		<0.0001
**Smoking status**						
Never smoker	239/3,604	7.3% (6.5–8.1)	76/1,233	7.8% (6.4–9.2)	163/2,371	7.2% (6.1–8.2)
Former smoker	39/300	9.7% (5.6–13.7)	29/237	10.6% (6.2–15.1)	10/63	3.2% (2.6–3.9)
Current smoker	86/1,063	12.0% (10.3–13.7)	82/945	11.6% (9.7–13.5)	4/118	12.1% (8.8–15.5)
*P for trend*		0.001		0.002		0.006
Cigarette smoking^*^, pack-year						
0	239/3,604	7.3% (6.5–8.1)	76/1,233	7.8% (6.4–9.2)	163/2,371	7.2% (6.1–8.2)
1–19	8/137	9.8% (5.8–13.9)	4/113	4.2% (0.8–7.6)	4/24	25.3% (9.0–41.7)
≥20	117/1,216	10.4% (8.7–12.2)	107/1,060	11.2% (9.3–13.1)	10/156	5.0% (1.0–8.9)
*P for trend*		0.002		0.001		0.165
Household air pollution^*^						
Yes	288/3,370	9.0% (8.0–10.0)	145/1,506	10.7% (9.1–12.3)	143/1,864	7.7% (6.5–9.0)
No	76/1,582	6.2% (5.1–7.2)	42/899	6.4% (4.9–7.8)	34/683	5.6% (3.9–7.3)
*P for difference*		<0.0001		<0.0001		0.018
**History of tuberculosis**						
Yes	37/254	11.6% (6.9–16.3)	18/119	12.8% (5.8–19.8)	19/135	10.0% (3.7–16.4)
No	327/4,713	7.8% (7.0–8.5)	169/2,296	8.9% (7.8–10.0)	158/2,417	6.8% (5.8–7.8)
*P for difference*		<0.0001		0.002		0.001
Exposure in the workplace^*^						
Yes	15/138	8.2% (2.7–13.7)	9/72	9.9% (1.7–18.1)	6/66	5.5% (3.3–7.6)
No	346/4,791	8.2% (7.4–8.9)	176/2,325	3.8% (2.4–5.2)	170/2,466	7.1% (6.1–8.2)
*P for difference*		0.105		0.123		0.489

*COPD, chronic obstructive pulmonary disease*.

* *Data missing for cigarette smoking (n = 10), household air pollution (n = 15) and exposure in the workplace (n = 38)*.

**Table 3:** Distributions of typical symptoms in the normal population and COPD patients by GOLD grades.

**Symptoms**	**Status**	**Number (%)**				
		**Normal**	**GOLD I**	**GOLD II**	**GOLD III-IV**	**Overall COPD**
Frequent cough	With	617 (13.6%)	53 (23.0%)	35 (31.0%)	7 (38.9%)	95 (26.3%)
	Without	3,907 (86.4%)	177 (77.0%)	78 (69.0%)	11 (61.1%)	266 (73.7%)
Sputum	With	572 (12.7%)	43 (18.7%)	34 (30.1%)	7 (38.9%)	84 (23.3%)
	Without	3,947 (87.3%)	187 (81.3%)	79 (69.9%)	11 (61.1%)	277 (76.7%)
Recurrent wheezing	With	221 (4.9%)	17 (7.4%)	17 (15.0%)	2 (11.1%)	36 (10.0%)
	Without	4,297 (95.1%)	213 (92.6%)	96 (85.5%)	16 (88.9%)	325 (90.0%)
Dyspnea in daily life	With	1,254 (27.7%)	104 (45.2%)	39 (34.5%)	8 (44.4%)	151 (41.8%)
	Without	3,271 (72.3%)	126 (54.8%)	74 (65.5%)	10 (55.6%)	210 (58.2%)
At least one of the above	With	1,771 (39.1%)	133 (57.8%)	64 (56.6%)	12 (66.7%)	209 (57.9%)
	Without	2,755 (60.9%)	97 (42.2%)	49 (43.4%)	6 (33.3%)	152 (41.8%)
CAT score	<10	1,045 (23.2%)	29 (12.6%)	17 (15.0%)	0	46 (12.8%)
	≥10	3,462 (76.8%)	201 (87.4%)	96 (85.0%)	17 (100%)	314 (87.2%)

*GOLD stage I (mild COPD): FEV1 ≥80% predicted; GOLD stage II (moderate COPD): FEV1 ≥50% to <80% predicted; GOLD stage III (severe COPD): FEV1 ≥30% to <50% predicted; GOLD stage IV (very severe); GOLD stage III–IV were combined because of small numbers. GOLD, Global Initiative for Chronic Obstructive Lung Disease; COPD, chronic obstructive pulmonary disease; CAT, COPD assessment test*.

In a multivariable-adjusted analysis, being aged ≥40 years, living at a lower altitude, being exposed to household air pollution, having a lower educational level and a history of tuberculosis were significantly associated with a higher prevalence of COPD (Table 4).

**Table 4:** Univariable-adjusted and multivariable-adjusted odds ratios of chronic obstructive pulmonary disease associated with risk factors.

**Variables**	**Univariable-adjusted**		**Multivariable-adjusted**	
	**OR (95% CI)**	***P*-value**	**OR (95% CI)**	***P*-value**
Male gender	1.12 (0.91–1.39)	0.275	1.25 (0.96–1.62)	0.095
**Age, years**				
15–39	1.00 (Reference)		1.00 (Reference)	
≥40	3.03 (2.40–3.82)	<0.0001	2.25 (1.72–2.95)	<0.0001
**Altitude,m**				
2,100–3,000	1.00 (Reference)		1.00 (Reference)	
3,000–4,000	0.57 (0.44–0.73)	<0.0001	0.61 (0.46–0.79)	<0.0001
>4,000	0.46 (0.35–0.62)	<0.0001	0.45 (0.33–0.61)	<0.0001
**Education level**				
Primary school and lower	1.00 (Reference)		1.00 (Reference)	
Middle and high school	0.38 (0.28–0.52)	<0.0001	0.49 (0.35–0.68)	<0.0001
College and higher	0.38 (0.27–0.53)	<0.0001	0.64 (0.43–0.95)	0.025
**Pack–years of smoking**				
0	1.00 (Reference)		1.00 (Reference)	
1–19	0.87 (0.42–1.80)	0.714	1.22 (0.58–2.57)	0.609
≥20	1.49 (1.18–1.88)	0.001	1.21 (0.91–1.60)	0.191
Household air pollution	1.82 (1.42–2.40)	<0.0001	1.34 (1.01–1.79)	0.043
History of tuberculosis	2.28 (1.58–3.29)	<0.0001	1.79 (1.23–2.61)	0.030
Exposure to dust or chemicals in the workplace	1.56 (0.90–2.70)	0.108	1.19 (0.67–2.10)	0.558

**Figure 3:**
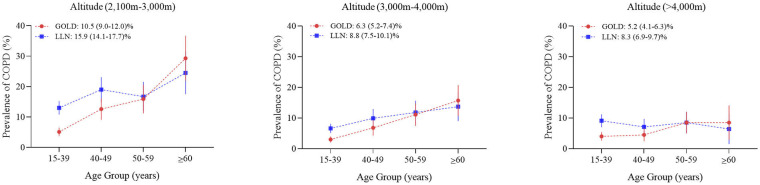
Prevalence of COPD using Lower Limit of Normal or GOLD criteria by age group and altitude of residence.

We also performed a sensitivity analysis using age-specific LLN to define COPD. The overall prevalence of LLN-defined COPD was 11.0% (95% CI 10.1–11.9%): 10.5% (95% CI 9.3–11.7%) in males and 11.4% (95% CI 10.2–12.6%) in females. The prevalence of LLN-defined COPD by altitude was shown in Figure 3.

## Discussion

This spirometry-based study in a representative sample of 4,967 residents shows that COPD is a major public health challenge at high altitudes (2,100–4,700 m): 8.2% of the high-altitude population aged more than 15 years had spirometry-defined COPD, making up for the gaps of region, altitude, and nationality. It also reveals that the majority of patients with COPD were unaware of their condition and that none of them had undergone previous pulmonary function testing. Finally, our data highlights exposure to household air pollution, history of pulmonary tuberculosis, and a low education level as major preventable risk factors for COPD in the high-altitude indigenous population.

Several cross-sectional surveys have reported the prevalence of COPD in various regions of the world below 1,500 m. The prevalence of spirometry-defined COPD in China in adults ≥40 years old is about 13.0%, with a 67% increase between 2004 and 2013 (6, 20, 21). In the BOLD study, the prevalence of COPD in 12 different sites worldwide was 10.1% for GOLD stage II or greater (11.8% for men and 8.5% for women) (22). In the US, the prevalence of COPD in 2010 was 14.0% (23). Data from Mongolia, Kazakhstan and Japan report prevalence rates between 6.7 and 13.6% (24–26). However, very limited specific data exist concerning prevalence of COPD in high altitudes.

There are a few prior studies on prevalence of COPD at high altitude. Data from the Peruvian Altiplano (3,825 m above sea level) report a prevalence of COPD of 6.1% in Puno, and 9.9% in the rural Puno area (27). Estimated prevalence of COPD in Mexico city (2,240 m above sea level) (Latin American Project for the Investigation of Obstructive Lung Disease (PLATINO) study) was 7.8% [95% CI 5.9%−9.7%] (28). In the PREPOCOL study, prevalence of COPD in Bogotá (Columbia, 2,640 m) was 8.5% (29). Data from 27 Mexican cities located at >2,000 m above sea level yield a prevalence of 15.6% (30). However, these studies used smaller sample sizes, incorporated no post-bronchodilator testing, or were restricted to local occupational or urban cohorts. Furthermore, albeit for the data from the Peruvian altiplano, these studies included mainly subjects living at lower altitudes than the high-altitude population described in this study. The present study is to our knowledge the only one to describe the overall prevalence of spirometry-defined COPD among high altitude residents using a rigorous sampling design and quality control and also, the first time, residents over 4,000 m above sea level were included.

Studies about the relationship between the prevalence of COPD and altitude show conflicting results. The PREPOCOL study, conducted in four Peruvian regions, and studies from Columbia and Kyrgyztan found a higher prevalence of COPD at high-altitude (29–31). In a systematic review and meta-analysis of 10 studies analyzing the prevalence of COPD at high altitude, Xiong H et al. found that results differed according to continents studied, prevalence of COPD at high altitude being higher in Asia (Kyrgystan) than in Europe (Austria) or Latin America (Mexico, Colombia, Peru). However, altitude was not found to be an independent risk factor for developing COPD (32). Conversely, in the PLATINO study performed in five major Latin America cities [Sao Paulo (altitude: 800 m), Santiago (543 m), Mexico City (2,240 m), Montevideo (35 m), and Caracas (950 m)] COPD prevalence was lowest in Mexico City, and there was a clear negative adjusted correlation between prevalence and altitude (28). Another study covering 27 Mexican cities (altitude range: 1–2,680 m) found a weak but significant negative correlation between altitude and COPD prevalence (30). Our study also suggests that the prevalence of COPD decreases as altitude increases. Among possible explanations are changes in lung volumes as a physiological adaptation to altitude: we noted slightly higher values for FEV_1_ and FVC (absolute and percent predicted) at higher altitudes, as well as an increase in FEV_1_/FVC. In addition, the proportion of heavy smokers decreased with increasing altitude. Also, proportion of people exposed to HAP and with a history of TB was lower in high altitude areas. These factors may be contributive to the decrease of COPD prevalence at higher altitude.

Household Air Pollution (HAP) mainly refers to coal and biomass fuels (e.g., animal dung, wood, crop residues) used indoors, also known as solid fuels (33). Increasing evidence suggests that exposure to HAP leads to increased incidence of COPD (34, 35). In 2015, HAP was estimated to be responsible for 2.9 million deaths and 85.6 million lost years of healthy life (36). The proportion of subjects exposed to HAP in our study (67.8%) was higher than that reported in a previous nationwide survey of COPD in China (26.7%). The present data also showed an increase of 36% (OR: 1.36) with HAP, confirming the increasingly acknowledged impact of HAP on respiratory health. Given the high prevalence of HAP and COPD, prevention of HAP and use of alternative fuels are important strategies for reducing COPD, especially for high altitude regions. Systematic reviews have suggested that HAP leads to significant risk for COPD in women (37–39). Cooking and heating with solid fuels more than 6 months leads to significant level of lung damage. Lung pathological findings in HAP-exposed individuals demonstrate small airways fibrosis and emphysema. The lung damage mostly occurs in women, as women often prepare the meals close to the fire in poorly ventilated houses for several hours daily. The present study also showed that women were more exposed to household air pollution than men (73.0% of women vs. 62.4% of men), a potential explanation for the lack of gender difference in the prevalence of COPD in this population.

Cigarette smoking is the major risk factor for COPD. Surprisingly, our multivariable analyses found no relationship between smoking and COPD after adjusting for gender, age, altitude, educational level, exposure to household air pollution, and history of tuberculosis. This is probably explained by a much lower prevalence of smoking than exposure to household air pollution (27.4 vs. 67.8%). However, as expected, prevalence COPD of smokers, especially heavy smokers, is higher than in non-smokers. Smoking prevention and cessation remain among the major important strategies for reducing COPD.

Incidence of tuberculosis remains high in many low and middle-income countries. An estimated 10 million new onset cases of TB and 1.57 million TB-related deaths occurred in 2017 (40). Tuberculosis is a possibly neglected risk factor for COPD in developing countries. In high-altitude areas, our study found 254 subjects (5.1%) with a history of tuberculosis, which is about 9-fold the prevalence in other provinces of China (0.6%) (6). Tuberculosis is known as a cause, comorbidity, and aggravating factor for COPD, as mentioned in the 2017 GOLD guidelines (41). The BOLD study found that a self-reported history of tuberculosis was significantly associated with airflow obstruction and spirometry restriction (42). We also found a significant association between a positive history of tuberculosis and the presence of COPD in participants aged ≥15 years (OR 1.77 [95% CI 1.21–2.58]. Thus, effective measures for tuberculosis prevention are an important component for the prevention of COPD in this population.

In our study, 56, 39, and 5% of patients with COPD had mild, moderate, and severe or very severe disease, respectively. This distribution was similar to reports in the USA and in China (20, 23). Although around two-thirds of patients with COPD were asymptomatic below 1,500 m sea level, our findings show 41.8% of the high-altitude COPD population without at least one respiratory symptom. In our study, only 1.1% of COPD patients were previously aware of their diagnosis. High rates of unawareness of COPD at high altitudes may be influenced by low access to health care, low index of suspicion by local physicians, poor access to spirometry, and by the fact that these subjects may tend to report significantly fewer respiratory symptoms. Individuals with undiagnosed COPD, whether they were asymptomatic or symptomatic, have an increased risk of acute exacerbations, pneumonia, and death. The US Preventive Services Task Force (USPSTF) recommends against screening for COPD in asymptomatic adults because of scant evidence showing a benefit of early detection and treatment (43, 44). These findings highlight the real challenges of COPD detection, and prevention in the high-altitude areas, especially for those asymptomatic subjects.

One major finding of this study is the risk factors affect a different degree to the prevalence of COPD at high altitude. Household air pollution may be the most important factors for COPD patients, provided more useful information for policymakers to consider more stringent household air pollution control measures. Another advantage is that the study reveals that the respiratory symptoms of people in high altitude are obviously affected by altitude factors, so lung function is particularly important for COPD screening. Final strength is our data suggest that the prevalence of COPD decreases as altitude increases, although previous publications provide conflicting results. Our study findings have important public health implications for residents at high altitude. The harsh high-elevation environment and backward economy are very challenging for the provision of healthy living conditions. Increased exposure to household air pollution and high rate of tuberculosis aggravate the hazards to pulmonary health. Our results call for an increased focus on COPD awareness, diagnosis, and treatment for residents living at high altitude. Controlling household air pollution via the replacement of fuel sources, initiating programs for smoking cessation and progress in health services for tuberculosis prevention should be considered public health priorities.

Our study has several limitations. First, smoking status was self-reported and not validated biochemically using exhaled carbon monoxide or urinary cotinine. Secondly, outdoor PM_2.5_ (particulate matter) was not analyzed in our study. We surmised that air quality at high altitudes has little effect on COPD prevalence because the outdoor PM_2.5_ in Lhasa was 26 ug/m^3^ from June 2015 to August 2016, much less than the national average level (45). Thirdly, we cannot eliminate the possibility that we misclassified people with asthma and other obstructive lung disorders as having COPD. Fourthly, the data covering subjects over 40 years of age were below estimated sample size.

In summary, our study documents the prevalence of COPD among a large sample of high altitude residents aged ≥15 years living at high altitude. Prevalence of COPD was inversely correlated with altitude. Furthermore, awareness of COPD was extremely low in this population. Increasing knowledge about COPD is important in this group. Household air pollution, a history of pulmonary tuberculosis and educational level were identified as major preventable risk factors for COPD.

## Data Availability Statement

The raw data supporting the conclusions of this article will be made available by the authors, without undue reservation.

## Ethics Statement

Written informed consent was obtained from the individual(s), and minor(s)' legal guardian/next of kin, for the publication of any potentially identifiable images or data included in this article.

## Author Contributions

YG, CW, and ZW conceived and designed the study. YG and CW supervised the study. SW, WM, and GS did the statistical analysis. YG and ZX drafted the manuscript. ZW, SW, and WM provide advices in the study and are listed in the acknowledgments. All authors contributed to acquisition, analysis, or interpretation of data, revised the report, and approved the final version before submission.

## Conflict of Interest

The authors declare that the research was conducted in the absence of any commercial or financial relationships that could be construed as a potential conflict of interest.
