# Supplementary material for: Prevalence and Risk Factors for COPD at High Altitude: A Large Cross-Sectional Survey of Subjects Living Between 2,100–4,700 m Above Sea Level
Source: Front Med (Lausanne). 2020 Dec 3;7:581763. doi: 10.3389/fmed.2020.581763 (PMC7744817; doi:10.3389/fmed.2020.581763)
Supplement: Supplementary file 1 [file Data_Sheet_1.pdf]

Figure E1. Flowchart and process of the population sampling.

1. Eight districts or counties were selected in both urban (2 districts) and rural (6 counties) areas of Tibet and Xinjiang using the probability proportional to size method
2. Two streets and townships were selected with the simple random sampling method from each district and county, respectively.
3. Three communities or village communities were selected using simple random sampling method from each of the street or township, respectively.
4. Participants from each of the sex/age strata from communities or villages were chosen using the simple random sampling method. Only one participant was selected from each household.

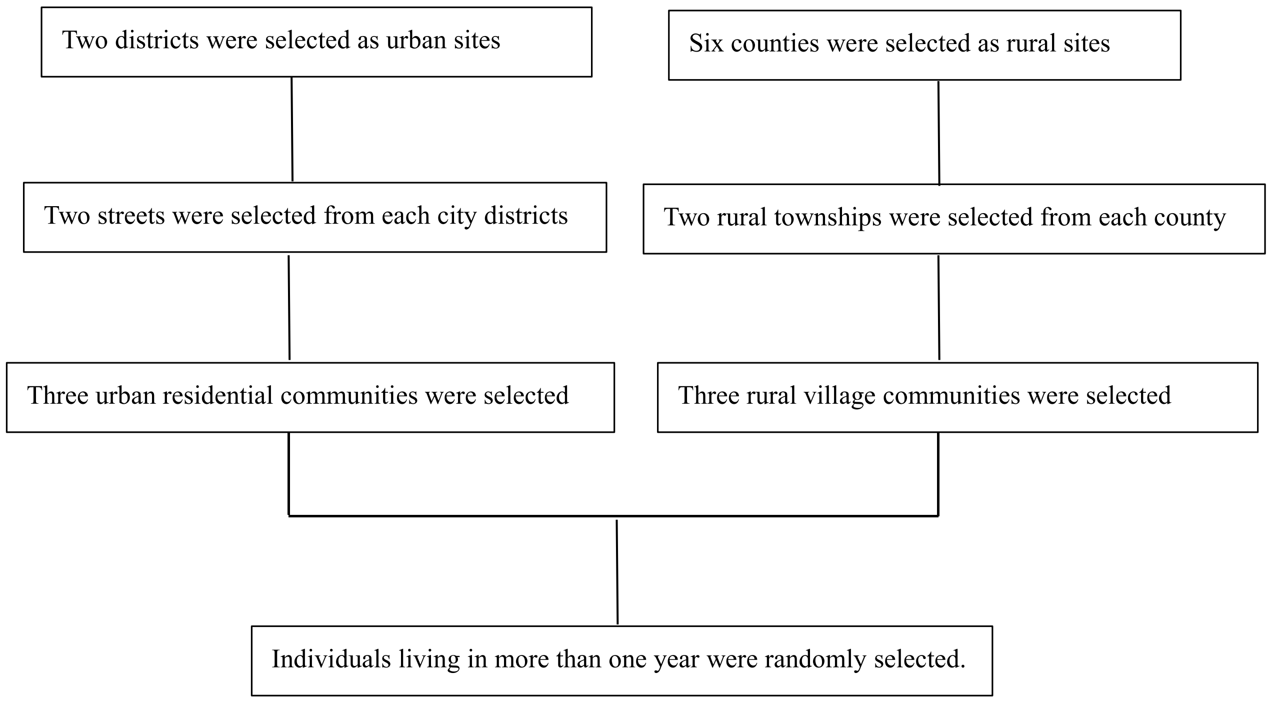

Figure E2. Sampling locations in the survey.

Lhasa Chengguan District (altitude 3,650 m above sea level, n=733) and Shigatse City (altitude 3,900 m, n=715), the only two cities in Tibet, were selected as urban sites. Six rural townships included: Linzhi County (altitude 3,000 m, n=808), Anduo County (4,700 m, n=613), Xietongmen County (4,100 m, n=232), Duilongdeqing County (4,500 m, n=601), Aheqi County (2,100 m, n=818) and Tashkurgan County (3,200 m, n=447)

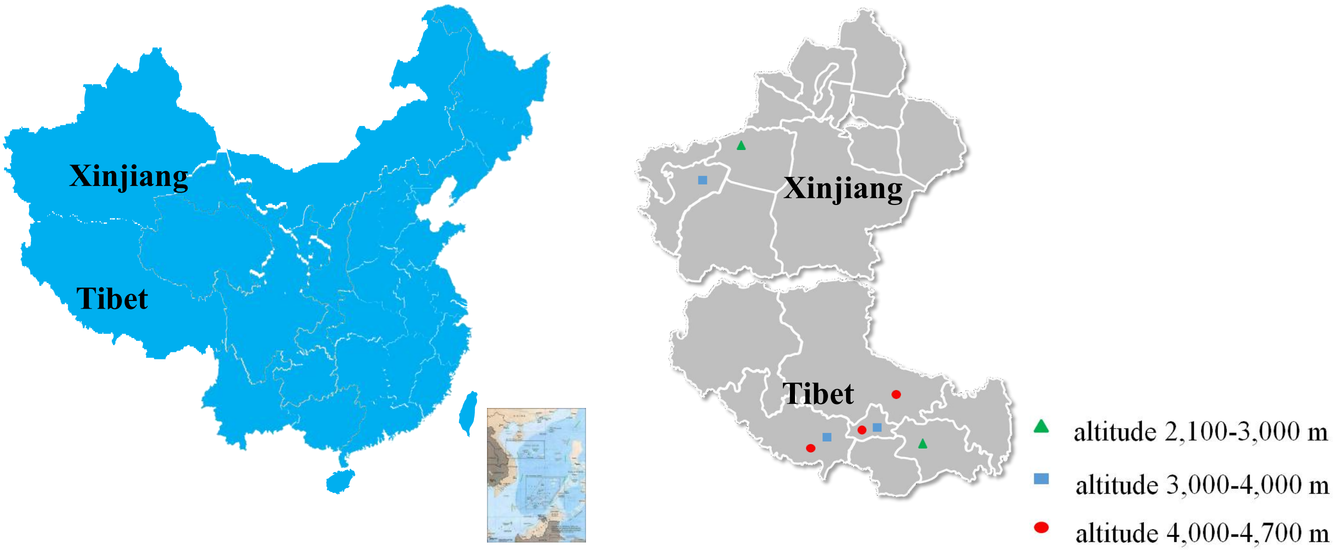

Table E1. Severity of COPD according to GOLD criteria at different altitudes of residence.

	**Overall**		**2,100-3,000m**		**3,000-4,000m**		**>4,000m**	
	**Cases (n)**	**Proportion (95%CI)**	**Cases (n)**	**Proportion (95%CI)**	**Cases (n)**	**Proportion (95%CI)**	**Cases (n)**	**Proportion (95%CI)**
**Mild**	232	63.7(58.8-68.7)	104	61.2(53.8-68.6)	75	63.0(54.2-71.8)	53	70.7(60.1-81.2)
**Moderate**	114	31.3(26.5-36.1)	58	34.1(26.9-41.3)	37	31.1(22.7-39.5)	19	25.3(15.3-35.4)
**Severe**	18	4.9(2.7-7.2)	8	4.7(1.5-7.9)	7	5.9(1.6-10.2)	3	4.0(0.5-8.5)

GOLD stage I (mild COPD): FEV1 ≥80% predicted; GOLD stage II (moderate COPD): FEV1 ≥50% to <80% predicted; GOLD stage III (severe COPD): FEV1 ≥30% to <50% predicted; GOLD stage IV (very severe); GOLD stage III–IV were combined because of small numbers. GOLD: Global Initiative for Chronic Obstructive Lung Disease. COPD: chronic obstructive pulmonary disease
